# Independent Evidence for the Preservation of Endogenous Bone Biochemistry in a Specimen of *Tyrannosaurus rex*

**DOI:** 10.3390/biology12020264

**Published:** 2023-02-07

**Authors:** Jennifer Anné, Aurore Canoville, Nicholas P. Edwards, Mary H. Schweitzer, Lindsay E. Zanno

**Affiliations:** 1 The Children’s Museum of Indianapolis, Indianapolis, IN 46208, USA; 2 Stiftung Schloss Friedenstein Gotha, 99867 Gotha, Germany; 3 Stanford Synchrotron Radiation Light Source, SLAC National Accelerator Laboratory, Menlo Park, CA 94025, USA; 4 Department of Biological Sciences, Campus Box 7617, North Carolina State University, Raleigh, NC 27695, USA; 5 Paleontology, North Carolina Museum of Natural Sciences, 11 W. Jones St., Raleigh, NC 27601, USA; 6 Department of Geology, Lund University, Sölvegatan 12, 223 62 Lund, Sweden

**Keywords:** synchrotron, bone remodeling, elemental analysis, molecular paleontology, diagenetic alteration

## Abstract

**Simple Summary:**

Our understanding of what can preserve in the fossil record, and for how long, is constantly evolving with the use of new scientific techniques and exceptional fossil discoveries. In this study, we examine the state of preservation of a *Tyrannosaurus rex* that died about 66 million years ago. This specimen has previously been studied using a number of advanced methods, all of which have indicated preservation of original soft tissues and bone biomolecules. Here, we use synchrotron—a type of particle accelerator—analyses to generate data identifying and quantifying elements that constitute this fossil bone. We show that trace elements incorporated by the living animal during bone deposition and remodeling, such as zinc, are preserved in the fossil bone in a pattern similar to what is seen in modern bird bones. This pattern is not observed in a microscopically well preserved, but molecularly more degraded dinosaur, a herbivorous *Tenontosaurus.* These data further support the preservation of original biological material in this *T. rex*, suggesting new possibilities for deciphering extinct species life histories. This study also highlights that preservation of original biochemistry in fossils is specimen-specific and cannot be determined by pristine appearance alone.

**Abstract:**

Biomolecules preserved in deep time have potential to shed light on major evolutionary questions, driving the search for new and more rigorous methods to detect them. Despite the increasing body of evidence from a wide variety of new, high resolution/high sensitivity analytical techniques, this research is commonly met with skepticism, as the long standing dogma persists that such preservation in very deep time (>1 Ma) is unlikely. The Late Cretaceous dinosaur *Tyrannosaurus rex* (MOR 1125) has been shown, through multiple biochemical studies, to preserve original bone chemistry. Here, we provide additional, independent support that deep time bimolecular preservation is possible. We use synchrotron X-ray fluorescence imaging (XRF) and X-ray absorption spectroscopy (XAS) to investigate a section from the femur of this dinosaur, and demonstrate preservation of elements (S, Ca, and Zn) associated with bone remodeling and redeposition. We then compare these data to the bone of an extant dinosaur (bird), as well as a second non-avian dinosaur, *Tenontosaurus tilletti* (OMNH 34784) that did not preserve any sign of original biochemistry. Our data indicate that MOR 1125 bone cortices have similar bone elemental distributions to that of an extant bird, which supports preservation of original endogenous chemistry in this specimen.

## 1. Introduction

The recent application of chemical and molecular techniques in paleontological research has resulted in a re-examination of the preservation potential of original biological chemistry in deep time. Such studies document the preservation of organic molecules and structures, ranging, for example, from elemental and microstructural evidence of color to ancient endogenous proteins, e.g., [1,2,3,4,5]. These discoveries push the boundaries of what was thought (or assumed) to preserve in the fossil record and have caused paleontologists to reconsider the canonical narrative that all original material is replaced during the fossilization process. Nonetheless, extraordinary claims require extraordinary evidence, and published studies documenting the preservation of organic molecules in fossils (particularly Paleozoic and Mesozoic fossils [1,2,3,4,5,6,7,8,9,10,11,12,13,14,15,16,17,18,19,20,21,22,23,24,25]) have, not surprisingly, been met with skepticism, e.g., [26,27,28]. One mechanism for increasing confidence in previous claims of endogenous chemistry in ancient fossils is the application of multiple and independent techniques to retest previous results in a non-destructive manner. Here, we review prior molecular studies, conducted over almost 20 years by multiple investigators, on *Tyrannosaurus rex* skeletal elements (two tibiae and one femur; Museum of the Rockies; MOR 1125) recovered from the Upper Cretaceous Hell Creek Formation of Montana, and use synchrotron X-ray fluorescence (XRF) to further test claims of original biochemistry preservation and limited diagenetic alteration in this specimen. We then contrast data collected from MOR 1125 with those collected from the tibia of the ornithopod *Tenontosaurus tilletti* OMNH 34784 from the Lower Cretaceous Cloverly Formation, and an extant avian radius (*Cacatua moluccensis*; NCSM 17977) showing similar cortical bone tissues.

### 1.1. Previous Research on MOR 1125

The *T. rex* femur of MOR 1125, was first studied in 2005 for signs of possible medullary bone [25], a sex-specific, estrogen-sensitive, ephemeral bone tissue produced by extant birds in lay [29]. This tissue identification was based on similarities in microstructure and location in both mineralized and demineralized tissues, when compared to those of extant laying ratites (ostrich and emu; [25]). Since then, 20 different techniques have been applied to this specimen, ranging from mass spectrometry to immunohistochemistry using antibody–antigen recognition (Table 1). Results from these studies showed exceptional micromorphological preservation extending to the cellular level (eg. osteocytes, possible endothelial cells; ([17,18,22,23,24,25], Schweitzer et al. in prep) as well as several biochemical signatures of bone-specific proteins (e.g., collagen; [17,18,19,23]). Additionally, these studies confirmed the observed molecular signatures differed from various diagenetically-induced morphologies/chemistries (e.g., biofilms; [14]) and were comparable to similar morphologies/chemistries of extant archosaurs. A summary of all molecular/chemical techniques applied to specimen MOR 1125 and the results obtained is provided in Table 1.

Accumulated data from multiple studies point to an endogenous source for these molecular signals. Here, we add to this body of evidence by applying new, highly sensitive and high-resolution methods to fully characterize the organic remains within this specimen. In this study, we apply Synchrotron XRF and XAS to further test the hypothesis that original biochemistry is preserved in this specimen.

### 1.2. Synchrotron XRF and XAS

Synchrotron radiation has many advantages over commercial X-ray analyses based on X-ray tubes, such as being monochromatic, high flux, and tunable [30]. These properties result in high sensitivity to dilute elemental concentrations (1 ppm) and the ability to significantly reduce data acquisition time. Additionally, samples can be analyzed under ambient atmosphere and temperatures, with no special preparation requirements. In some synchrotron XRF imaging stations, decimeter scale samples can also be accommodated, which removes the need for subsampling (e.g., [4]).

Monochromatic X-rays allow the selection of a very narrow bandwidth of X-ray energy/wavelengths which facilitates X-ray absorption spectroscopy (XAS). XAS is one of the most common and most powerful techniques at synchrotron sources as it provides information about the atomic structure of the absorbing atom, allowing the determination of elemental speciation [30]. In the case of studying fossil tissues, this information is critical in determining whether a detected element is derived from organic or inorganic processes. Furthermore, XRF imaging and XAS can be combined to produce maps of elemental species, which adds a further layer of our capability to tease out physiological processes from diagenetic ones in extinct organisms.

### 1.3. Previous Synchrotron Work on Fossils

Synchrotron XRF and XAS has been used to examine and interpret the biochemistry of fossils for over 10 years, in specimens recovered from a wide range of geologic ages (~400 mya to recent), tissue types (non-biomineralized (e.g., skin) and mineralized (e.g., bones, teeth) and taxa (invertebrates, vertebrates and plants) [4,8,10,11,12,15,16,31,32,33,34]. These studies revealed biological structures that cannot be observed in visible light, as well as the fractionation of elements within discrete biological structures that can be compared with similar tissues in living organisms. Resulting data have led to the identification of specific elemental biomarkers for a number of biosynthetic pathways, including those involved in bone remodeling, repair and deposition [10,11,16,31,32,33,34].

In previous synchrotron analyses involving bone, zinc (Zn) and, in most cases, strontium (Sr), were found to correlate within areas of active ossification, including fracture calli, growth plates and around secondary osteons [10,11,16,32,33,34,35,36,37,38,39,40]. Sr was also shown to be correlated to diagenetic processes, being isolated to the Haversian canal, and was differentiated from organically-derived Sr through differences in distribution patterns and chemical coordination [11]. Calcium (Ca) distributions were found to be similar to Zn and concentrated in areas of ossification as well as the cutting cones of secondary osteons [10,11,16,32]. Although distributions and concentrations varied slightly depending on species, these patterns were seen in both extant and extinct vertebrates, with the oldest preservation of these patterns seen in a 150 mya dinosaur phalanx [16].

### 1.4. Bone Elemental Biomarkers

In extant vertebrates, elemental biomarkers of bone physiology are most often concentrated on areas of active ossification [32,33,34,35,36,37,38,39,40]. Zn is one of these, because it plays a structural role when incorporated into the hydroxyapatite (HAP) lattice, forming Zn–Fluorine (F) complexes [35,39]. Zn is also critical for osteoblastogenesis, stimulating bone formation and inhibiting bone resorption [35,39]. The expression of Zn has been found to be highest in osteocytes, which express alkaline phosphatase (ALP) and osteocalcin (OCN) in mineralized tissue [36]. Elevated concentrations of Zn localize to zones between mineralized and unmineralized tissue within osteons, suggesting a role in bone mineralization and cellular regulation [34,40]. Finally, Zn is associated with matrix metalloproteinases (MMPs), which are important for cartilage degradation [41]. In fracture healing, Zn is expressed between the first and second week in rats, where it increases the expression of OCN needed for hard callus formation [35].

Other key elements in bone physiology include sulfur (S), phosphorus (P), Ca and Sr. Sulfur comprises both the organic and inorganic constituents of bone, aiding in both collagen (e.g., cysteine and methionine; [42]) and HAP structure (sulfate; [43]). Sulfur is also an important constituent of glycoproteins found in various skeletal tissues types (e.g., keratan sulfate in medullary bone and other fast growing skeletal tissues; [13,44]). Sr increases osteoblast activity by increasing osteoblastic marker expression in ALPs, bone sialoprotein (BSP), and OCN, all of which affect osteoblast proliferation and differentiation [37,38]. It also interferes with osteoclast dissolution of bone mineral, by disrupting the actin cytoskeleton organized in the sealing zone, a thick band of actin needed for osteoclast apical-basal polarization and resorption [37].

Ca is most associated with bone formation and metabolism, with a majority of vertebrate Ca found in the HAP structure of bones and teeth [44]. In the skeleton, Ca bound in apatite ([Ca_3_(PO_4_)_2_]_3_Ca(OH)_2_) lattice serves as structural support (rigidity, strength and elasticity) and as a reservoir for Ca needed for other metabolic processes throughout the body. Like Ca, P is predominantly found in bone and teeth [45]. It is primarily associated with Ca, both in HAP and amorphous calcium phosphate. Inorganic P is one of the most important components for HAP formation during the mineralization of the extracellular matrix [45].

### 1.5. Elements Associated with Fossilization and Diagenetic Alteration

Elements observed in fossilized skeletons are either endogenous, or diagenetic, the latter being influenced by the depositional environment. For fossils from the Hell Creek Formation, the most common and abundant diagenetic element seen in fossil specimens is iron (Fe). In some specimens, the amount of Fe is so high that it masks any possible biological signatures [Anné, unpublished work]. In other specimens, Fe is constrained to the mineral infill of the fossil cavities (e.g., medullary cavity and vascular spaces). Other elements usually associated with diagenetic alteration or mineral infill are silicon (Si) and Ca, derived from the various silicate and carbonate minerals associated with fossilized bone ([10,16,33]; Anné, unpublished work).

Here, we test the hypothesis that the biochemical signatures detectable in MOR 1125 are endogenous, and were present in the once-living animal. We predict that if original biochemistry is preserved in MOR 1125, then the distributional patterns of elements crucial to bone formation and remodeling will mirror those found in extant bone, with elevated and localized concentrations of Ca, Zn and possibly S and Sr as described above.

We also compared results from MOR 1125 to extant bone, as a positive control, and to those we derived from the Cretaceous dinosaur *Tenontosaurus tilleti* (OMNH 34784). The latter introduces taxonomic and depositional variables, allowing us to compare the preservation potential between two fossil bones that differ in geological age and depositional environments.

## 2. Materials and Methods

### 2.1. Biologic Samples

**Figure 1:**
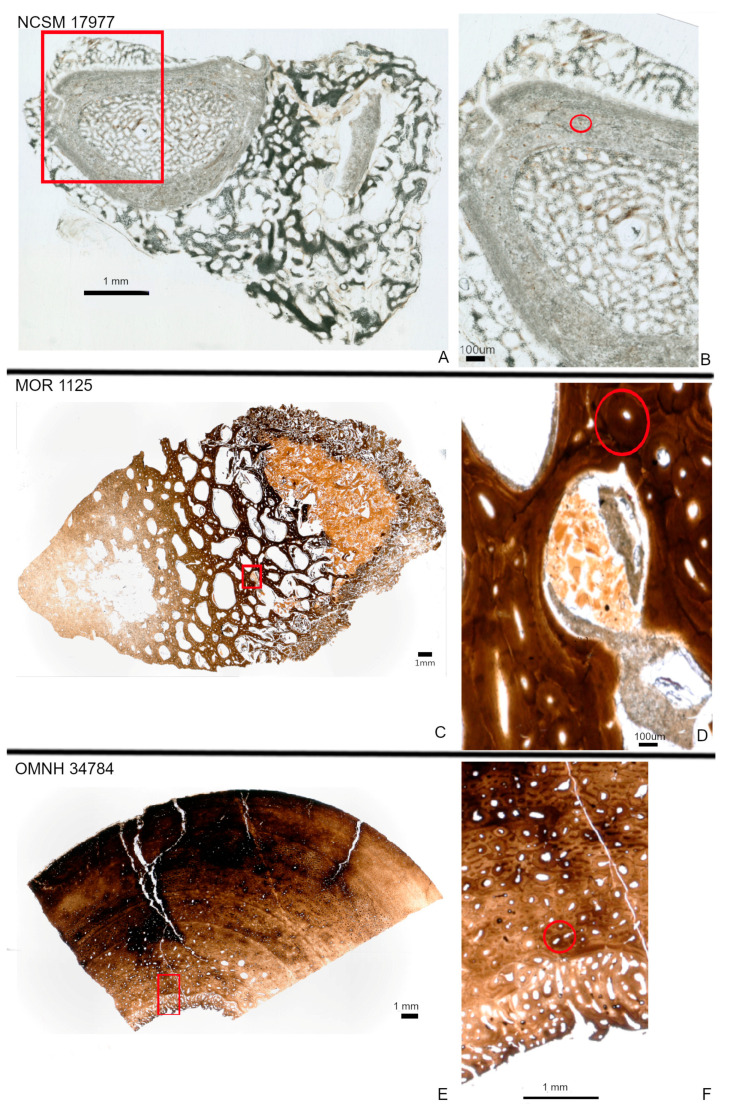
Histology of NCSM 17977 (radius; (**A**,**B**)), MOR 1125 (femur fragment; (**C**,**D**)) and OMNH 34784 (tibia fragment; **E**,**F**). Red boxes indicate where areas of higher magnification were taken. Areas of higher magnification represent those areas scanned for synchrotron XRF. NCSM 17977 is represented by a completed transverse section (**A**). The normal radius wall is composed of cortical bone with large secondary osteons (highlighted with red circle; (**B**)). Pathological tissues on both the periosteal and endosteal surfaces consist of trabeculae. The femur fragment of MOR 1125 consists of a dense Haversian tissues identified by multigenerational secondary osteons (highlighted with red circle) and trabeculae, with crushed bone between some trabeculae (**D**). OMNH 34784 consists of both a “normal” cortex and an unusual endosteal tissue deposited along the endosteal margin and filling part of the medullary cavity (**E**). The cortex is comprised of fibrolamellar bone that is more remodeled towards the endosteal margin as seen by multigenerational secondary osteons (highlighted with red circle; (**F**)).

Our extant bone sample (NCSM 17977) derives from the radius of the bird *Cacatua moluccensis*. This specimen bears mostly remodeled cortical tissue encased by a fracture callus and reactive bone tissue from a possible infection (Figure 1A,B). The “normal” cortical bone of the radius is mostly composed of secondary tissue with large secondary osteons. The fracture callus is represented by trabecular tissue on both the periosteal and endosteal surfaces. The endosteal pathological tissue completely fills the medullary cavity, while the periosteal tissue more than doubles the diameter of the element (Figure 1A,B).

Fossils are represented by a femoral fragment of *Tyrannosaurus rex* (MOR 1125) recovered from the lower Hell Creek Formation (~67–68 my [46]) and a tibial fragment of *Tenontosaurus tilletti* OMNH 34784 from the Cloverly Formation (~124–98 my [47]). Both specimens have been previously studied histologically [24,48,49] and both were described as containing an unusual endosteal tissue consistent with reproductive (“avian”) medullary bone [29]. The bone fragment of MOR 1125 considered in this study consists of portions of the deep cortex adjacent to this endosteal spongious tissue. The deep cortex consists mostly of a dense Haversian tissue with several generations of secondary osteons. The associated spongiosa is formed of secondary trabeculae (Figure 1C,D). Between some trabeculae, there is a fine and crushed bone tissue that is similar in location and microstructure to reproductive medullary bone (Figure 1D) and previous molecular studies supported such identification [13,25].

The OMNH 34784 tibial fragment we analyzed is composed of fibrolamellar bone with secondary remodeling marked by several secondary osteons in the deep cortex, some of which are overlapping (Figure 1E,F). Radially-oriented endosteal trabecular bone is present within the medullary cavity. This tissue has been interpreted as potential medullary bone [48].

### 2.2. Synchrotron Analyses

XRF and XAS were performed at the Stanford Synchrotron Radiation Lightsource (SSRL) at beam lines 2-3, 7-2 and 14-3 (specifics for each beam line listed below). At these beam lines, XRF imaging is performed by mounting the sample on high precision encoded stages and rastering the sample relative to the incident X-ray beam in a continuous scan mode at a 45° angle to the incident beam. The XRF signal is detected with a Hitachi Vortex silicon drift diode detector positioned at 90° to the incident beam. The detector is coupled to a Quantum Detectors Xspress3 multi-channel analyzer system. Data collection is achieved by the stage moving in a continuous horizontal motion, with an image pixel defined by the cumulative XRF counts binned as a function of distance traveled (step size) and velocity over this distance (dwell time). At the end of each horizontal motion, data acquisition stops, the data is read from the detector system, and the stages move to the beginning of the next line. The fluorescence energies for 16 user selectable elements are collected, as well as the full XRF spectrum per pixel. The 16 elements can be displayed live during data collection and are shown here. The full XRF spectrum data is used in cases where deeper interrogation of the XRF data is required. Further details on signal processing of the imaging system are provided in [50,51]. XAS spectra were collected in fluorescence mode. XRF image data were processed using the MicroAnalysis Toolkit software and XAS spectra were processed using the SIXpack [52,53].

#### 2.2.1. Beam Line 7-2

Beam line 7-2 is a hard X-ray wiggler beam line optimized for XRF imaging and XAS of high *Z* elements (Ca and heavier). It has an energy range of ~5–16 keV with a Si(111) monochromator. The X-ray spot size on the sample is achieved with XOS polycapillary focusing optics with a ~35 µm or ~65 µm spot size available. In this study, an incident beam energy of 11 keV and a spot size of 35 µm was used. XRF signal was detected with a 4 element Hitachi Vortex silicon drift diode detector. 7-2 has a regular scan range of ~400 × 300 mm which allows for multiple samples (such as thin sections) to be mounted simultaneously or can accommodate larger scale samples.

#### 2.2.2. Beam Line 2-3

Beam line 2-3 is a hard X-ray bending magnet beam line optimized for XRF imaging and XAS of high *Z* elements (Ca and higher) at a higher resolution than 7-2 (but smaller scan range, 25 × 25 mm). It has an energy range of ~5–19 keV with a Si(111) monochromator. The X-ray spot size on the sample is achieved with Sigray axially symmetric focusing optics with 5 µm or 1 µm available. In this study, an incident energy of 13.5 keV and a spot size of 5 µm was used. XRF signal was detected with a 1 element Hitachi Vortex silicon drift diode detector.

#### 2.2.3. Beam Line 14-3

Beam line 14-3 is a tender X-ray bending magnet beamline optimized for XRF imaging and XAS of low *Z* elements (S, P, Si, Cl). It has an energy range of 2.1–5 keV with two Si(111) monochromators (phi 0 or phi 90). The X-ray spot size on the sample is achieved with Sigray axially symmetric focusing optics with 5 µm or 1 µm available. In this study, a spot size of 5 µm was used. The sample is placed in a helium purged atmosphere to minimize X-ray attenuation from air. In this study, XRF signal was detected with a 4 element Hitachi Vortex silicon drift diode detector in earlier experiments, but in later experiments the system was upgraded to a 7 element detector.

**Figure 2:**
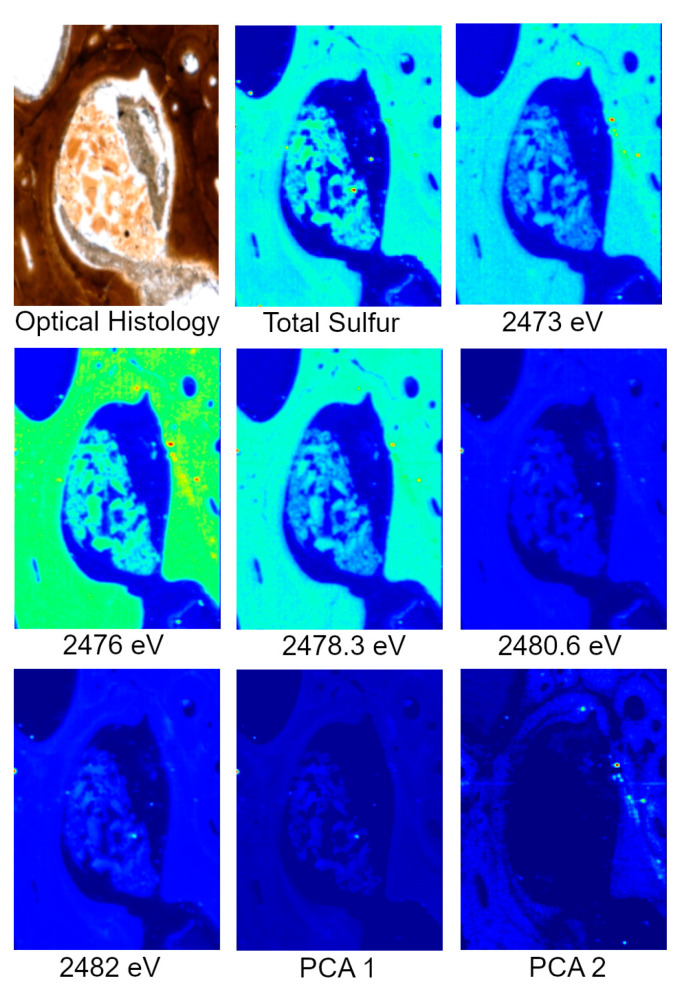
Optical histology, total S, S species, and PCA of S species maps from MOR 1125 (*T. rex*). Optical histology identifies tissues of interest for scanning, in this case Haversian tissue (remodeling) and possible medullary bone (reproductive). Total S represents the distribution of all S species within the area of interest. Various S maps are taken at known energies of important S species within bone, both organic and inorganic. PCA analyses show the greatest difference between the maps, usually indicating differences in concentration and species. XANES are taken based on highlighted areas of species differences in PCA 2 to identify exact S species.

Specimens analyzed at beamline 14-3 were first scanned at 2500 eV to detect a range of elements. For these maps, only total S can be visualized. To identify variations in S species, several scans were acquired at energies that correlate peaks in the XAS spectra known to be characteristic of specific S species, organic and inorganic, associated with the matrix of interest, in this case, bone. For this study, those energies correlate to ~2473, ~2476, ~2478, ~2480 and ~2482 eV. Principle Component Analysis (PCA) of the images is then performed which produces images that assist in highlighting the distribution of S species. These PCA images are then used to select target locations for S XANES, which are then used to identify the specific S species. An example of the step by step process from total S to S XANES is highlighted for MOR 1125 in Figure 2.

## 3. Results

### 3.1. XRF Imaging

**Figure 3:**
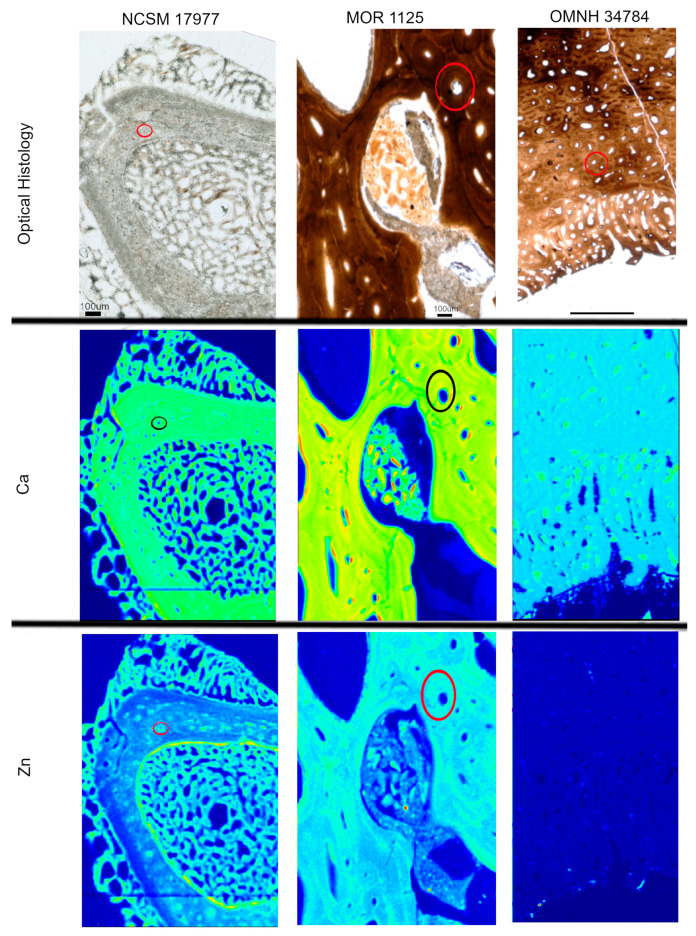
Optical histology and XRF maps taken from beamline 2-3 of Ca and Zn taken for all specimens. Warmer colors correspond to higher concentrations. Secondary osteons are highlighted in Ca and Zn (example osteon highlighted by circles) in NCSM 17977 and MOR 1125. OMNH 34784 shows uniform distribution of Zn, with concentrated Ca in pores between tissues.

**Figure 4:**
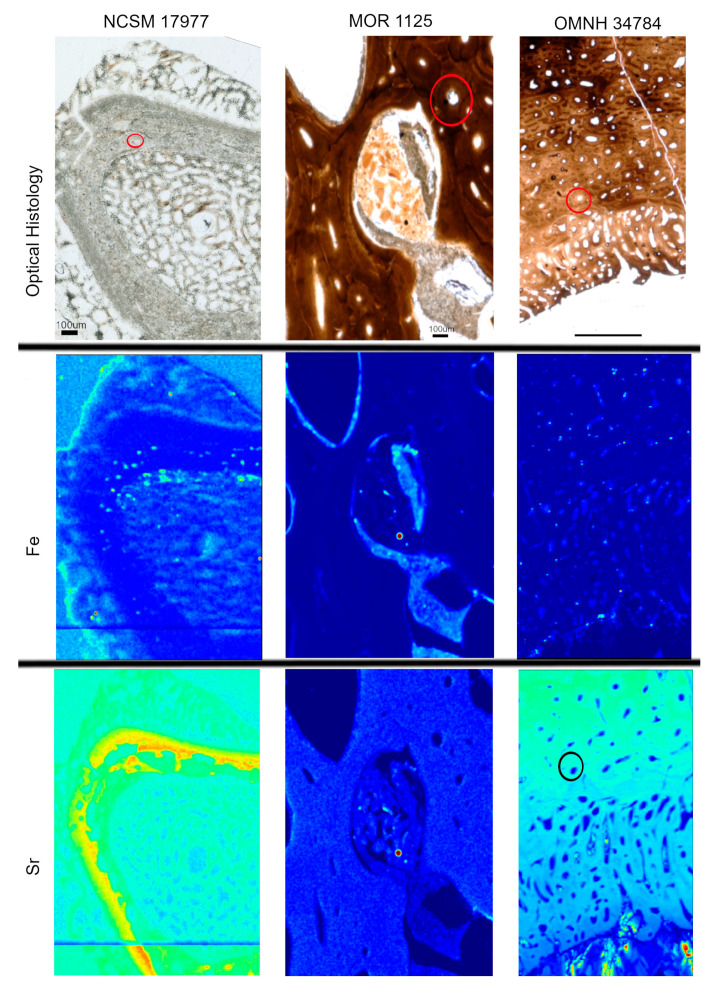
Optical histology and XRF maps taken from beamline 2-3 of Fe and Sr for all specimens. Warmer colors correspond to higher concentrations. Fe is concentrated in spaces between pathologic tissues in NCSM 17977 and within areas of infill between tissues in MOR 1125 and OMNH 34784. Sr is elevated in thicker cortical bone versus the thin trabeculae of the pathologic tissues in NCSM 17977. Sr is relatively uniform in MOR 1125. In OMNH 34784, secondary osteons are highlighted in Sr (example osteon circled).

Heterogeneous distributions of the elements S, Ca and Zn were observed within discrete histological features of the extant sample, NCSM 17997 (Figure 1 and Figure 3). Ca and Zn highlight areas of remodeling, with elevated concentrations (rings of brighter areas) associated with secondary osteons (Figure 1 and Figure 3). Zn is also elevated within the pathological tissues. Fe is concentrated in spaces between the trabeculae of the pathologic tissues. Sr is elevated in the thicker cortical tissue compared to the finer trabeculae present in pathologic tissues (Figure 4). PCA 2 of S species highlighted potential different species between normal cortical bone and the pathological tissues as well as at the periosteal surface (see XANES results).

Elemental distributions for MOR 1125 were similar to those seen in the extant sample, with Ca and Zn highlighting areas of remodeling (Figure 1 and Figure 3). Fe was highly localized to vascular spaces and along the edges of some bone trabeculae (where some sediment was still present); it was not detected in the adjacent fossil bone tissue, consistent with chemical sequestration and minimal diagenetic alteration of MOR 1125 bone tissue (Figure S1, Figure 1 and Figure 4). Differences in the distribution of S species between different bone tissues were highlighted by PCA of S species maps (Figure 2; see also XANES results). No other distinctions were seen in other elements (Figure S2).

In contrast to MOR 1125 (*T. rex*), OMNH 34784 (*T. tilletti*) displays uniform distributions of Ca and Zn in both cortical and endosteal bone, with higher concentration of Ca in some, but not all vascular canals, consistent with diagenetic deposition of calcite or another Ca-rich mineral in these areas (Figure 1 and Figure 3). Sr showed elevated concentrations in the cortical bone matrix relative to the endosteal bone (Figure 4), and is also present in lower concentrations in the secondary bone tissue forming some of the deep cortical secondary osteons, when compared to adjacent primary cortical tissue. Some bright spots of Sr are also visible within the medullary cavity. Fe is concentrated within the vascular canals and to some extent within the medullary cavity (Figure 4), similar to that seen in MOR 1125. PCA 2 of S species showed only differential distribution between the bone and diagenetic infill (see XANES results).

### 3.2. Zn XANES

**Figure 5:**
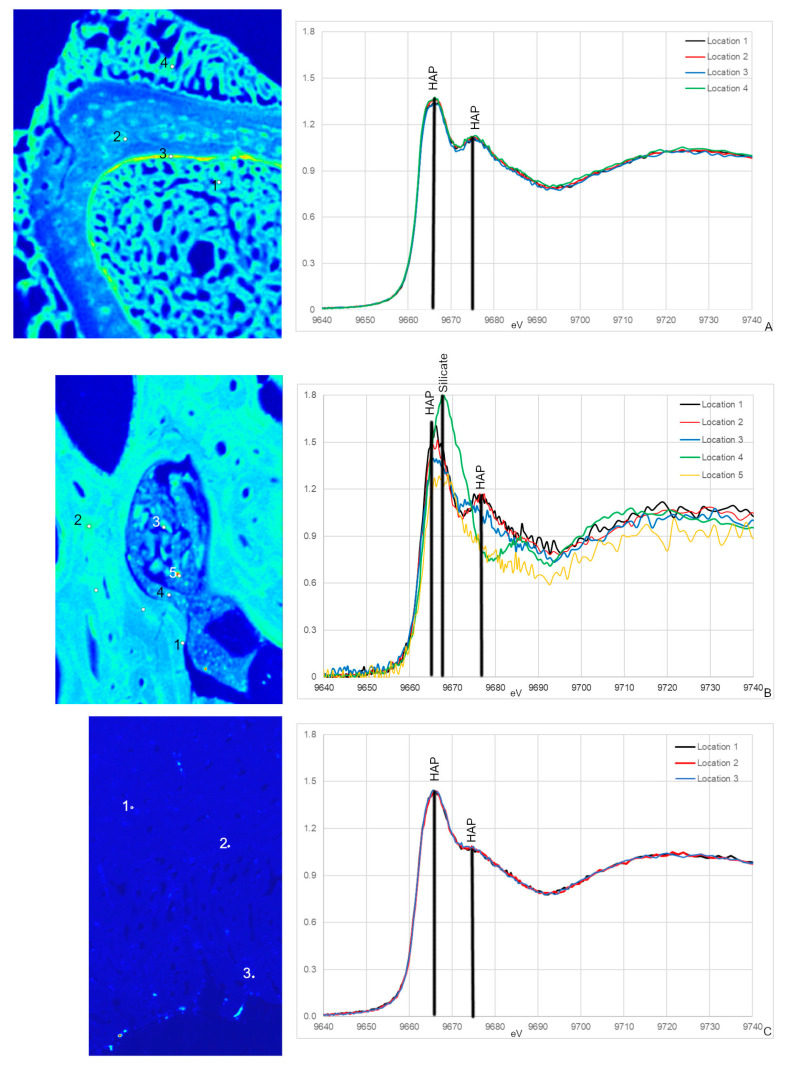
Zn XANES from NCSM 17977 (*C. moluccensis* radius; (**A**)), MOR 1125 (*T. rex* femur; (**B**)) and OMNH 34784 (*T. tilletti* tibia; (**C**)) and with peaks labeled using peak designations based on [54,55,56]. The locations for the XANES spectra are labeled on the corresponding Zn elemental maps. All spectra show peaks associated with hydroxyapatite (HAP) at ~9665 and ~9675 eV, respectively. For MOR 1125, an additional peak at 9678 eV in spectrum 4 is associated with silicates.

All Zn XANES spectra from NCSM 17977 (extant sample) exhibit peaks associated with HAP at ~9665 and ~9675 eV, with the most distinct peak at ~9675 eV (Figure 5A; [54,55,56]). These peaks were also observed in MOR 1125 with the exception of spectrum 4 (Figure 5B). Spectrum 4 was taken in a cavity containing sediments and smaller bone fragments and differs in several ways from all other spectra including unique peaks at 9668 eV, which is associated with some Zn silicates [54]. This is the only spectrum taken in an area that visually does not appear to be bone tissue, but rather mineral infill (Figure 5A). There is also a unique peak in spectra taken from cortical bone (spectra 1 and 2) that is not seen in the potential medullary bone tissue (3 and 5) at 9676 eV.

Zn XANES spectra from OMNH 34784 are similar and only exhibit peaks for HAP at ~9665 and ~9675 eV (5C). Both HAP peaks are broader compared to NCSM 17977 and MOR 1125, with the second peak at ~9675 eV reduced to almost a shoulder.

### 3.3. S XANES

**Figure 6:**
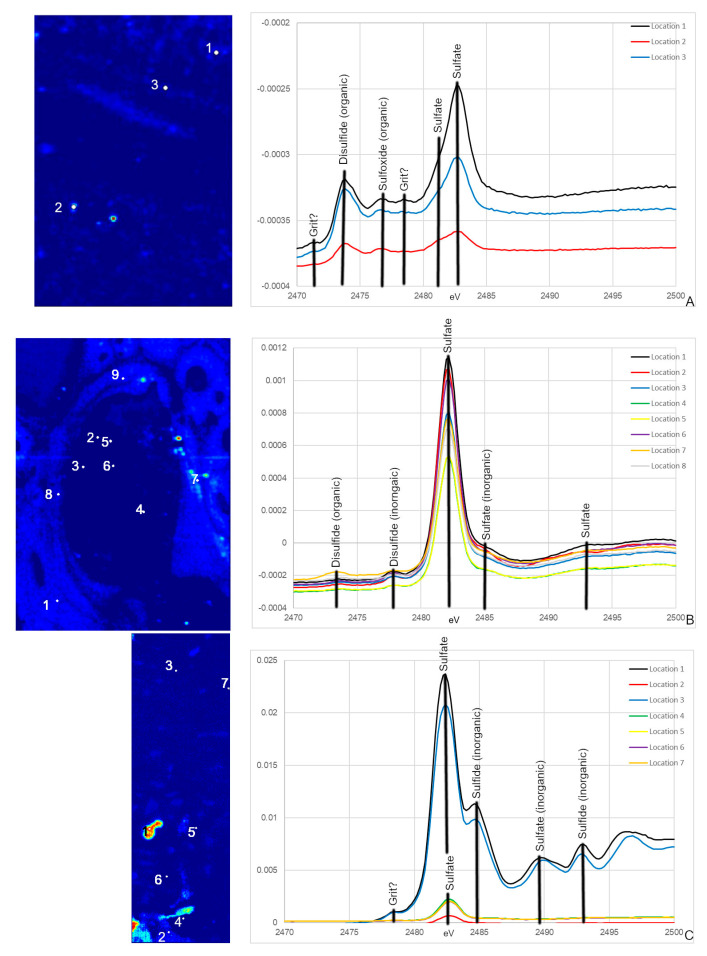
S XANES from NCSM 17977 (*C. moluccensis* radius; (**A**)), MOR 1125 (*T. rex* femur; (**B**)), OMN 34784 (*T. tilletti* tibia; (**C**)) with peaks labeled using peak designations based on [57,58]. The locations for the XANES spectra are labeled on the corresponding S PCA2 maps. Both NCSM 17977 and MOR 1125 exhibit organic disulfide and sulfate peaks. MOR 1125 and OMNH 34784 also have inorganic S peaks for disulfides, sulfates and for OMNH 34784, sulfides.

NCSM 17977 exhibits both organic and inorganic S species, with peaks associated with organic disulfides and organic and inorganic sulfides (Figure 6A; [57,58]). Many of these peaks are also seen in MOR 1125, including the disulfide peak at ~2473.5 eV and the sulfate peak at ~2482 eV (Figure 6B) thus consistent with an endogenous source in this dinosaur. NCSM 17977 shows additional organic S species peaks for sulfoxides, as well as some for Native S, which is possibly powder used for thin section grinding. Peak intensity is much lower in endosteal tissue, with broader peaks. OMNH 34784 S XANES does show the dominate sulfate peak at ~2482 eV (Figure 6C). However, all remaining peaks correlate to inorganic sulfates and sulfides, including peaks associated with Fe, Zn, Mg and silicates (Figure 6C).

## 4. Discussion

### 4.1. Evidence for Endogenous Biochemistry of MOR 1125

Synchrotron XRF imaging and XAS spectroscopy supports previous multidisciplinary studies showing evidence for retention of original biochemistry in the femur of MOR 1125 (*T. rex*). The distribution of elements Ca and Zn, as well as the suite of detected S species, and the correlation of these to specific histological features associated with bone remodeling, repair, and active ossification (Figure 1, Figure 2 and Figure 3) are similar to those seen in the extant bird, NCSM 17977, and support an endogenous source and cannot be readily explained by diagenetic overprint [10,11,16,31,32,33,34,59,60,61]. In contrast, despite exceptional histological preservation, OMNH 34784 results varied widely from the *T. rex* and extant bird specimens, showing more uniform distributions of Ca and Zn associated with diagenetic alteration, and a lack of organic S species such as the disulfide peak at ~2473.5 eV (Figure 1, Figure 3 and Figure 6).

The results of this study demonstrate the ability of synchrotron XRF imaging and XAS analyses to identify, spatially resolve, and characterize elements affiliated with discrete biochemical processes in various bone tissue types. This ability may be used in future studies as a way to distinguish between tissue types such as medullary versus pathologic tissues.

### 4.2. Endogenous Elements in MOR 1125- Ca, Zn and S

MOR 1125 showed Ca, Zn and S elemental distributions and species that correlated to biological processes in (Figure 2, Figure 3, Figure 5 and Figure 6). Elevated Ca within the secondary osteon radius may be due to osteoblastic regulation of mineralization during bone deposition, or by an increase of free Ca released from the bone during the resorption stage of remodeling [10,11,32]. Elevated Zn around secondary osteons has been demonstrated by others on analysis of human osteons, where Zn is known to pool in forming osteons, especially within osteoid at the mineralization front [34,35,36]. Both extant and fossil specimens exhibit the typical HAP peaks of bone tissue in Zn XANES at ~9665 and ~9675 eV (Figure 5, [54,55,56]).

Speciation maps of S revealed that different species within MOR 1125 are distributed differently in areas of remodeling and within endosteal (medullary-like) tissue (Figure 2 and Figure 6). Difference in S species within areas of remodeling may be due to S associated with bone mineral (e.g., inorganic and organic sulfate) versus bone collagen (e.g., organic disulfide). Sulfur XANES confirmed that both NCSM 17977 and MOR 1125 exhibit peaks that correspond to multiple organic species of S, notably disulfides and sulfates (Figure 6; ~2473 eV and ~2482 eV). Sulfate is found in the HAP structure of bone in both organic and inorganic phases [55,62]. Organic sulfate is also associated with keratan sulfate (KS), which is found in various skeletal tissues in low concentrations, but is also associated with the deposition of medullary bone in avians at much higher levels (e.g., [43]). MOR 1125 also exhibited peaks for organic disulfides at ~2473 eV [31,58]. Disulfides such as methionine have been found to preserve in association with osteons in archaeological and fossil bone [31].

### 4.3. Diagenesis in MOR 1125—Fe and S

These data, together with those from previous studies, strongly indicate that original chemistry is preserved in the bone of *T. rex* MOR 1125. However, this specimen also shows evidence of alteration. In both fossil specimens, elevated Fe is confined to diagenetic mineral infill areas between the bone tissue (Figure S1 and Figure 4) that correspond to regions where Fe can be introduced during fossilization (i.e., spaces in the medullary cavity, vascular canals). In addition to peaks associated with bone bioapatite, both MOR 1125 and OMNH 34784 exhibit inorganic S species peaks that correlate to geological input, including inorganic sulfides (e.g., pyrite), sulfates (e.g., gypsum) and sulfites (e.g., MgSO3; Figure 6). The mixture of organic and inorganic S species in MOR 1125 allows us to tease apart primary biochemistry from diagenetic influence in these specimens and supports our interpretation of only limited diagenesis in MOR 1125.

### 4.4. Differences in Preservation between Fossil Specimens

Despite a shared histological integrity, OMNH 34784 differs from MOR 1125 in the degree of chemical alteration, with little to no sign of endogenous material preserved. This fails to support the hypothesis that histological integrity can be directly correlated to organic preservation. None of the elemental distributions for OMNH 34784 overlap with MOR 1125 or extant material with the exception of the diagenetic Fe (Figure 2 and Figure 4). When comparing XANES, Zn XANES for OMNH 34784 did show the classic HAP peaks at ~9665 and ~9675 eV, though these peaks are broader in OMNH 34784 than in the other two specimens (Figure 5). Broadening may be due to a mixture of different Zn species or concentrations of Zn within the HAP structure, both which may be caused by diagenetic alteration [62]. For S XANES, OMNH 34784 lacks any species of organic S (Figure 6).

## 5. Conclusions

The XRF and XAS results of this study support previous findings on MOR 1125. Histological analyses, both traditional and CT, confirm excellent morphological preservation of bone tissue at the microstructural and nanostructural levels [24,25]. We then correlated these micromorphological patterns to obtained elemental maps, to show the specific biological features associated with bone remodeling, which has also been seen in extant organisms in both this study and others [10,11,16,31,32,33,34]. Synchrotron results strongly support previous studies claiming endogenous preservation at the molecular level, including immunohistolochemical studies confirming the presence of an original biomolecule signal in MOR 1125 and mapped these signals to skeletal regions (i.e., medullary bone). This presents promising implications for the use of synchrotron XRF to differentiate between pathological bone, and reproductive (medullary) bone in MOR 1125, specifically. Although some differences in element distribution are seen between MOR 1125 and the extant specimen, OMNH 34784 shows evidence of far greater chemical alteration, to the exclusion of endogenous material. This highlights that preservation of endogenous bone biochemistry in fossils is specimen-specific and likely tied to variation in depositional history and fossilization processes, although this relationship is far from being fully understood. We conclude that the lack of original molecular/chemical preservation in one specimen cannot be used to eliminate the possibility that it may be preserved in other specimens, regardless of age, type or morphology.

## Figures and Tables

**Table 1:** List of various techniques applied and results obtained from molecular/chemical studies of the *T. rex* MOR 1125.

Analytical Technique	MOR 1125 Tissue Type	Results and Citation
Transmitted light microscopy (LM)	Demineralized endosteal bone (MB) and cortical bone (CB)	Flexible, collagen-like fibrous matrix, transparent, hollow vessel-like structures, osteocyte-like microstructures with filopodia [24,25]
Scanning electron microscopy (SEM)	Demineralized Medullary Bone (MB) and Cortical Bone (CB)	Fibers arranged as bundles, consistent with collagen [24]
SEM	Isolated microstructures consistent with osteocytes (hereafter ‘osteocytes’ to conserve space) and microstructures consistent with blood vessels (hereafter vessels) liberated from demineralized CB	Isolated, free-floating 3-D osteocyte morphs, with long, extensive filipodia and intracellular contents, consistent in morphology and location to osteocytes from extant vertebrates; interconnected, hollow, flexible and transparent microstructures consistent with extant vessels [18,23,25]
SEM	Biofilm grown in extant deproteinated bone	Patchy distribution after demineralization of bone substrate, unable to maintain shape, differed morphologically from structures observed in MOR 1125; no visible osteocyte structures [14]
Atomic force microscopy (AFM)	Demineralized CB and MB	Matrix fibers demonstrating periodicity of ~70 nm consistent with collagen [22]
Transmitted electron microscopy (TEM), localized electron diffraction	Ultrathin sections of CB and MB	Mineral phase identified as biogenic hydroxylapatite (HA) [22]
Transmitted electron microscopy (TEM), localized electron diffraction	Isolated vessels and osteocyte liberated from CB	Iron intimately associated with vessel walls and osteocyte surfaces. Vessel walls under TEM retain differentiated structures and layers, consistent with extant vessels ([17,18,22,23], Schweitzer et al., in prep)
Electron energy loss spectroscopy (EELS)	Vessels and osteocytes liberated from demineralized CB	Iron localized to vascular walls and osteocytes, but not seen in fibrous matrix [17]
Micro-X-ray fluorescence mapping of Fe	Vessels liberated from demineralized CB	Confirmation of iron localizing to vessel walls [17]
Micro-X-ray Fe XANES	Vessels liberated from demineralized CB	Diagenetic goethite Fe within vessels and vessel walls, also seen in treated extant vessels [17]
Immunohistochemistry (IHC), antibodies against chicken collagen I (Col I) and osteocalcin (OC)	Demineralized CB and MB	Localized binding of antibodies to tissue, supporting preservation of Col I and OC epitopes, using multiple controls [23]
IHC to actin antibodies	Isolated vessels and osteocytes from demineralized CB	Localized antibody binding in patterns seen in extant homologues. Different binding patterns in each tissue/cell type support endogeneity, along with negative controls [17,18]
IHC using antibodies to chicken phosphoendopeptidase (PHEX)	Osteocytes liberated from demineralized CB	PHEX antibodies localized to osteocyte-like structures, but not to matrix. Binding was specific and controls were negative. Antibody specificity was confirmed to be avian specific; no binding was seen to extant crocodile osteocytes, only bird [18]
ICH using antibodies to DNA backbone	Osteocytes liberated from demineralized CB	Antibodies localized in a single spot internal to some dinosaur osteocytes. No binding was seen to filopodia or other materials [18]
IHC using antibodies to keratan sulfate	Demineralized CB and MB	Binding in a globular pattern in extant and dinosaur MB, no binding in adjacent CB from the same specimens [13]
Histochemical localization using DNA intercalating stains propidium iodide (PI) and 4′, 6′-diamidino-2-phenylindole dihydrochloride (DAPI)	Osteocytes liberated from demineralized CB	Binding followed pattern seen with anti-DNA backbone antibodies, specific to internal region of cell, and only a single spot. No staining was observed on filopodia or other structures [18]
Histochemical localization using Alcian blue.	Demineralized CB and MB	Differential staining, with MB much more intensely stained than CB, consistent with MB in extant avian bone [13]
Histochemical localization with High-Iron Diamine (HID)	Demineralized CB and MB	Differential staining, with MB staining much more intensely relative to CB, consistent with extant avians [13]
Time-of-flight secondary ion mass spectrometry (ToF-SIMS)	Demineralized CB and MB	Identification of amino acids glycine (gly) and alanine (ala) support presence of Col I [23]
Mass spectrometry (MS)	Demineralized and extracted vessels, matrix and osteocytes	Multiple collagen and actin peptide sequences [18,20,21]
Peptide mapping	Peptides recovered from demineralized and extracted bone matrix	Fossil-derived peptides mapped to monomers 2,3, and 4 on extant collagen models [19]
Laser ablation- inductively coupled plasma mass spectrometry (LAICP-MS)	Sectioned CB	Average concentration of exogenous elements from diagenesis (e.g., Fe) lower than in other Hell Creek specimens, with only moderate alteration [7]

## Data Availability

Data available on request due to restrictions.

## Supplementary Materials

The following supporting information can be downloaded at: https://www.mdpi.com/article/10.3390/biology12020264/s1, Figure S1 shows gross elemental maps for specimens MOR 1125 taken at beamline 7-2. Figure S2 shows additional XRF maps of MOR 1125 that did not show any correlations with specific histological features.
