# Supplementary figures and images for: Independent Evidence for the Preservation of Endogenous Bone Biochemistry in a Specimen of Tyrannosaurus rex
Source: Biology (Basel). 2023 Feb 7;12(2):264. doi: 10.3390/biology12020264 (PMC9953530; doi:10.3390/biology12020264)

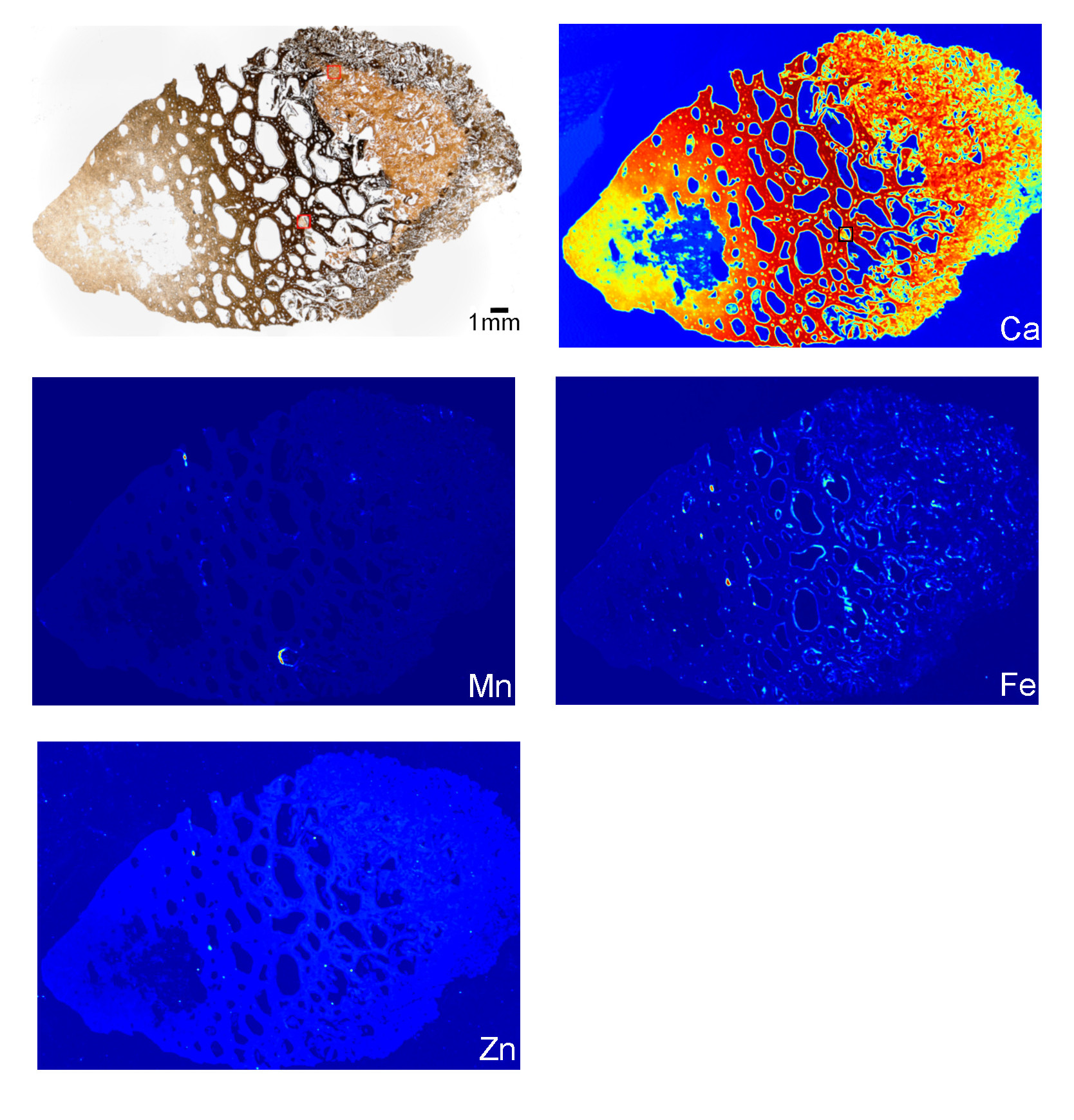

Supplement: Supplementary file 1 [file biology-12-00264-s001.zip › Figure S1 Gross elemental maps for specimens MOR 1125 taken at beamline 7-2.jpg]

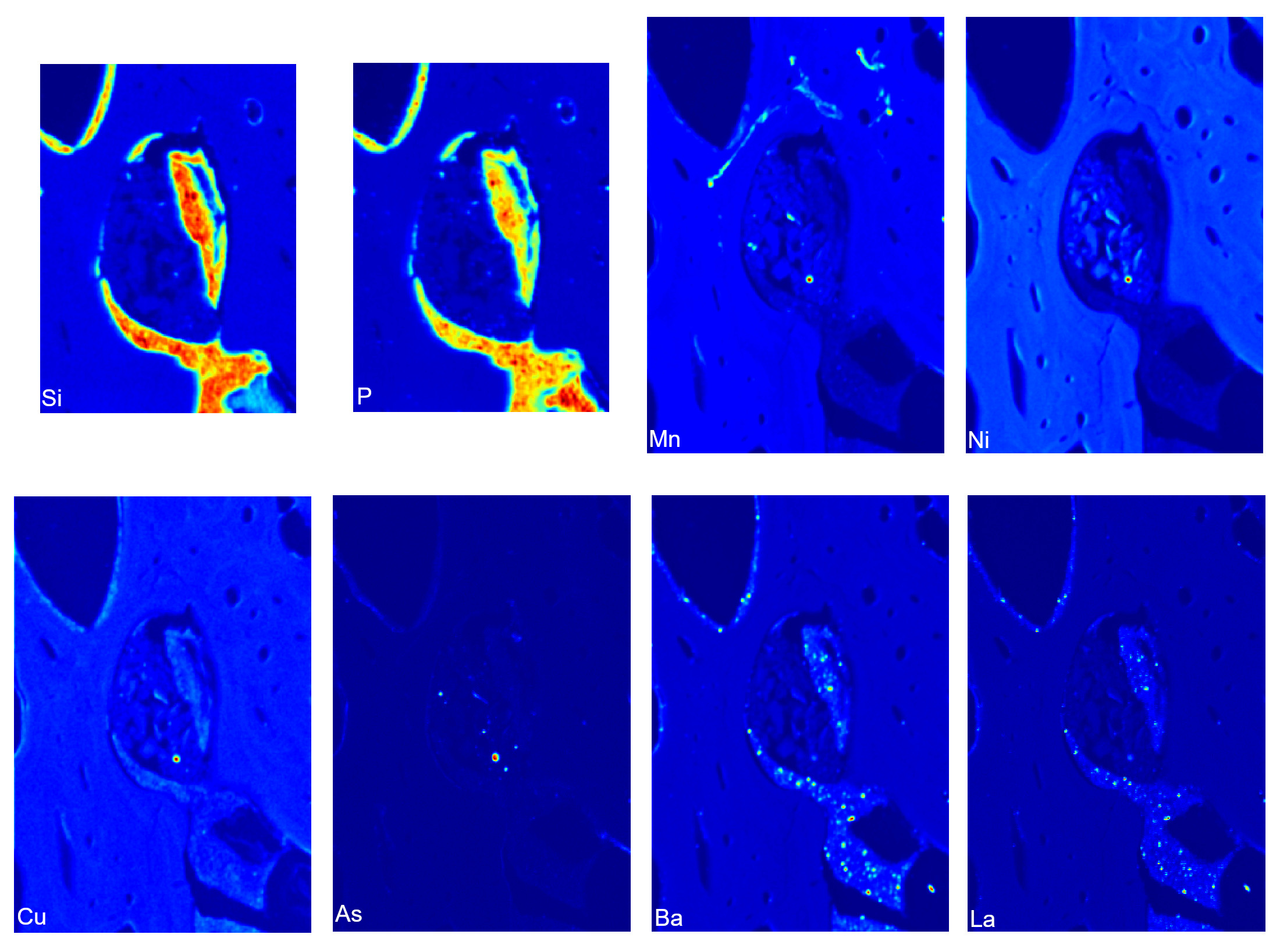

Supplement: Supplementary file 1 [file biology-12-00264-s001.zip › Figure S2 Additional XRF maps of MOR 1125 that did not show any correlations with specific histological features.jpg]
